# Comparison of molecular and microscopic technique for detection of *Theileria annulata* from the field cases of cattle

**DOI:** 10.14202/vetworld.2015.1370-1374

**Published:** 2015-11-28

**Authors:** H. C. Chauhan, B. K. Patel, A. G. Bhagat, M. V. Patel, S. I. Patel, S. H. Raval, H. H. Panchasara, M. D. Shrimali, A. C. Patel, B. S. Chandel

**Affiliations:** 1 Division of Animal Biotechnology, College of Veterinary Science & Animal Husbandry, Sardarkrushinagar Dantiwada Agricultural University, Sardarkrushinagar - 385 506, Gujarat, India; 2 Division of Veterinary Microbiology, College of Veterinary Science & Animal Husbandry, Sardarkrushinagar Dantiwada Agricultural University, Sardarkrushinagar - 385 506, Gujarat, India; 3 Division of Veterinary Pathology, College of Veterinary Science & Animal House, Sardarkrushinagar Dantiwada Agricultural University, Sardarkrushinagar - 385 506, Gujarat, India; 4 Teaching Veterinary Clinical Complex (College Clinics), College of Veterinary Science & Animal House, Sardarkrushinagar Dantiwada Agricultural University, Sardarkrushinagar - 385 506, Gujarat, India

**Keywords:** Giemsa staining, polymerase chain reaction, theileriosis

## Abstract

**Aim:**

Tropical theileriosis is fatal hemoprotozoal disease of dairy animals caused by *Theileria annulata*. The aim of the present study was to detect the *T. annulata* and comparison of results of molecular and microscopic techniques.

**Materials and Methods:**

A total of 52 blood samples were collected from the cattle suspected for theileriosis across the Banaskantha district. All the samples were screened for theileriosis using Giemsa’s staining technique and polymerase chain reaction (PCR).

**Results:**

Total of 17 (32.69%) and 24 (46.15%) samples were found positive for theileriosis by microscopic examination and PCR test, respectively. It revealed that the study area is endemic for theileriosis, and the microscopic technique has 70.83% sensitivity and 100% specificity with respect to PCR technique.

**Conclusion:**

It may be concluded from the present study that the PCR is comparatively sensitive technique than microscopic examination and may be recommended to use in the field for screening of theileriosis in the study area, where a high prevalence of diseases have been reported due to intensive dairy farming.

## Introduction

Theileriosis is a hemoprotozoan parasitic disease caused by *Theileria annulata* affects mainly crossbred cattle. The disease is transmitted mainly by ticks of the genus *Hyalomma* and characterized by lymphadenopathy, splenomegaly, fever, anemia, weakness, and loss of body weight [1,2]. It is an economically important tick-borne disease in tropical and sub-tropical regions including India [3]. In India, Gujarat is one of the leading states in milk production contributing about 10.32 million tons per year, having around 17 lakh exotic/crossbred milch cattle [4,5]. Rearing of crossbred cattle has been increased in Gujarat since last decade, which has resulted in to increased milk production of the state; however, it has also increased the incidence of various hemoprotozaoan infections. Theileriosis has become endemic in the state and prevalent as clinically inapparent/carrier, and may cause fatal disease [6], resulting in to substantial economic loss to the state. Therefore, early and specific diagnosis of disease is of paramount importance to reduce the economic loss as well as to maintain the good health status of the high milk producing animals.

The diagnosis of theileriosis is mainly based on clinical signs and confirmed by microscopic examination of Giemsa stained thin blood or lymph node smears for presence of piroplasms in red blood cells and macroschizonts in lymphocytes. Diagnosis based on the clinical signs is not trustful in many cases, as other parasitic diseases also show clinical signs similar to those of tropical theileriosis. Whereas, microscopic method of diagnosis demands expertise in slide reading for sub-clinical or chronic infections cases, as parasitemias in such cases are often extremely low and piroplasm may be difficult to find in stained blood smears. This conventional method is difficult to perform and time consuming to identify piroplasmic form within the erythrocytes from carrier animals [7]. Which ultimately increase the chances of getting false negative or false positive results [8]. Indirect immunofluorescent antibody test (IFAT) is OIE recommended test for diagnosis of parasitic diseases. It detects circulating antibodies against antigens of piroplasms and/or macroschizonts but the cross-reactivity with antibodies directed against other *Theileria* species limits the specificity of IFAT. Thus, false positive or negative results are frequently observed [7,9-11]. Moreover, antibodies tend to disappear in long-term carriers although *Theileria* piroplasms persist. Hence, animals with negative serological test may be positive for *T. annulata* piroplasms and can be act as a source of infection for susceptible animals. Furthermore, it is not applicable for the determination of pre-symptomatic or carrier animals, where parasitemia is very low [12]. Therefore, diagnostic method having high sensitivity and specificity than routinely using serological and microscopic examination is the need of the hour.

In the present study, molecular method (polymerase chain reaction [PCR]) has been developed to diagnose the theileriosis from the persistently infected cattle [13] and the prevalence of *T. annulata* was recorded using microscopic method and PCR.

## Materials and Methods

### Ethical approval

All samples were collected as per standard sample collection method without any stress/harm to animals.

### Collection of samples

Banaskantha district was selected as study area for present study. It lies on north-west side of Gujarat State, India between 23.33 to 24.45 north latitude and 72.15 to 73.87 east longitudes. Total 52 blood samples were collected in EDTA containing tubes from cattle of above 1 year age. The samples were collected from the animals which were showing symptoms like high fever, swelling of sub-mandibular and sub-scapular lymph nodes and hemoglobinuria or which have the problem of tick infestation. Thin blood smears were prepared from each sample at the site of sample collection, and remaining part of sample was brought to the laboratory over ice for further investigation.

### Microscopic examination

A thin blood smear from each sample was prepared and fixed in methanol for 5 min and stained with Giemsa stain for 30 min. Blood smears were examined for intraerythrocytic forms of *Theileria* spp. piroplasma under ×100 objective magnification. More than 20 microscopic fields per slide were observed before considering it as negative; whereas, even the presence of single piroplasms was recorded as positive for *Theileria*.

### DNA extraction

DNA was extracted from the blood samples using Qiamp DNA blood and tissue kit (Qiagen, Netherland) following the manufacture’s protocol. In brief, 20 µl of proteinase K was added into 2 ml of microcentrifuge tube containing 100 µl of anticoagulant treated blood. 200 µl of AL buffer was added into microcentrifuge tube. Mixture was thoroughly mixed by vortexing and incubated at 56°C for 10 min. Then 200 µl of ethanol was added and further mixture was thoroughly mixed by vortexing. Mixture was taken in DNeasy Mini spin column placed in a 2 ml collection tube. Mixture was centrifuge at 8000 rpm for 1 min and flow-through and collection tube were discarded. Spin column was placed in a new 2 ml collection tube. 500 µl of AW1 buffer was added and then again mixture was centrifuge at 8000 rpm for 1 min and flow-through and collection tube were discarded. Spin column was placed in a new 2 ml collection tube. 500 µl of AW2 buffer was added then mixture was centrifuge at 14,000 rpm for 3 min and flow-through and collection tube were discarded. Spin column was transferred to a new 2 ml microcentrifuge tube. DNA was eluted by adding 200 µl of AE buffer at the center of the spin column membrane. Mixture was incubated at room temperature (15-25°C) for 1 min and then mixture was centrifuged at 8000 rpm for 1 min.

### PCR

For detection of *T. annulata*, forward primer 5’- CCAGGACCACCCTCAAGTTC-3’ and reverse primer 5’- GCATCTAGTTCCTTGGCGGA-3’ were used, which amplify 430 bp fragment of the *Tams1* gene which encodes 30-kDa merozoitepiroplasm surface antigen of *T. annulata* [14]. PCR was carried out in 200 µl PCR tubes using Nexus gradient Master cycler (Eppendorf, Germany). Each 15 µl of the PCR mixture comprised of 1 µl of DNA, 7.5 µl of ×2 PCR master mix, 0.5 µl of each *Tams1* forward and reverse primer and 5.5 µl nuclease free water. The PCR conditions includes initial denaturation at 95°C for 5 min; followed by 30 cycles of 95°C for 30 s (denaturation), 55°C for 30 s (annealing) and 72°C for 30 s (extension); with a final extension step of 72°C for 4 min. 5 µl of amplified PCR product was mixed with 1 µl of ×6 gel loading dye and subjected to electrophoresis in 1.5% agarose gel along with 100 bp DNA ladder. The images were captured and documented using gel documentation system (Bio Rad., USA).

### Determination of sensitivity and specificity of microscopic examination

The sensitivity and specificity of microscopic method was determined in respect to PCR assay using following formula use by Noaman [15].


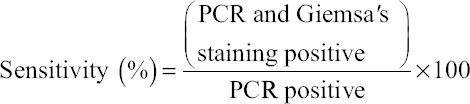



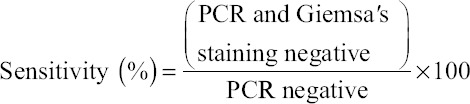


## Results

### Microscopic examination of blood smears

**Figure-1:**
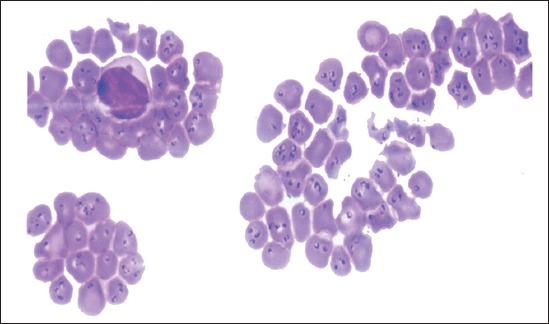
Blood smear stained with field stain showing the comma and signet-ring shaped intra-cytoplasmic bodied of *Theileria* organism (1000X magnification).

Out of 52 samples, 17 (32.69%) samples were found positive in Giemsa’s stained blood smear (Table-1). All the samples had showed comma and signet-ring (diameter of 0.5-1.5 µm) shaped intra-cytoplasmic bodied which was seems to be *Theileria* piroplasms (Figure-1). Sensitivity and specificity of microscopic examination was found to be 70.83% and 100%, respectively in respect to 100% PCR assay (Table-2).

**Table-1:** Comparison of results of microscopic examination and PCR assay for detection of *T. annulata* from the field samples.

Method/technique	Results of microscopic examination technique	

	Positive	Negative
Results of PCR assay		
Positive	17	07
Negative	00	28

PCR=Polymerase chain reaction, *T. annulata=Theileria annulata*

**Table-2:** Sensitivity and specificity of microscopic method in respect to PCR assay for detection of *T. annulata* from the field samples.

Method/technique	Number of samples examined	Number of positive samples	Sensitivity (%)	Specificity (%)
Microscopy	52	17	70.83	100
PCR	52	24	100	100

PCR=Polymerase chain reaction, *T. annulata=Theileria annulata*

### PCR

**Figure-2:**
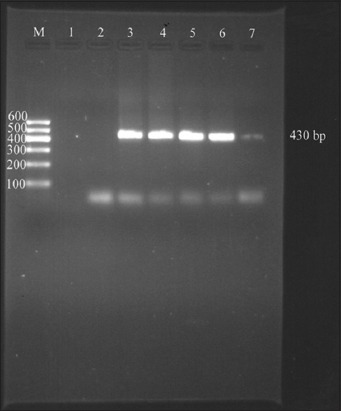
Agarose gel electrophoresis showing the 430 bp PCR product. M: 100 bp molecular wt. DNA marker, Lane 1: Negative control, Lane 2: Negative sample, Lane 3 – 7: Positive samples showing specific amplicon of 430 bp size.

On agarose gel electrophoresis of PCR products, 24 (46.15%) samples showed specific amplification for *T. annulata* corresponding to 430 bp (Figure-2).

### Treatment

All the theileriosis positive cattle detected by PCR were treated with single dose of buparvaquone (2.5 mg/kg body weight) and hematinic (feritas at 10 ml I/M). Out of 24 theileriosis positive cattle, 21 (87.5%) cattle was respond to the treatment and recovered in 4-5 days.

## Discussions

*T. annulata* is a causative agent of tropical theileriosis, is an omnipresent parasite in India and has been reported from every part of the country. The disease is important in India and it was estimated that 250 million cattle in countries including Iran, Turkey, India and China are at a risk of the disease. It causes serious economic loss to the economy of various developing countries in terms of loss of livestock and production [16,17]. In recent time, production of milk has been increased in Banaskantha district, Gujarat. Eventually, rearing of Holstein Friesian, Jersy and their crossbred animals have been increased. Therefore, prevalence of hemoprotozoal diseases like theileriosis and babesiosis is increased, which causes high mortality and leads to huge economic losses.

The present study envisaged the appraisal of diagnosing theileriosis in dairy crossbred cattle using microscopic slide examination as well as PCR. The sensitivity and specificity of each technique were also compared simultaneously. On examination of 52 samples, it was revealed that microscopic examination has 70.83% and 100% sensitivity and specificity, respectively, which corroborates the observation made by Noaman [15]. In the present study, less sensitivity of microscopic method observed which might be due to artifacts, low parasitemia, and destruction of piroplasmic forms in red blood cells because of hemolysis, thickness and dirtiness of slides. Less sensitivity of microscopic examination has also been reported by other workers [18,19]. The present study showed 32.69% (17/52) and 46.15% (24/52) prevalence of disease based on microscopic examination and PCR, respectively. The high prevalence of theileriosis observed in the present study may be attributed due to high abundance of tick vector, because the hot and humid environment is very conducive for ticks and ultimately for the survival of the piroplasm. Previous workers has also reported the high sensitivity of PCR, which supports our findings that the modern techniques like PCR is comparatively more sensitive and specific than routine microscopic examination [7,15-17,20].

Microscopic examination of Giemsa stained blood smear is routinely used for diagnosis of theileriosis worldwide including India, because it is simple to perform, quick and cost effective techniques. However, less sensitivity makes it difficult to detect carrier cases [21]. Similarly, serological test has also limited value because antibodies specific to theileriosis disappear rapidly, and may give false negative results in case of carrier animals. Detection of carrier animal has special value in control program for theileriosis, as these animals pose a risk for maintenance and perpetuation of *Theileria* in environment through animal-tick-animal cycle. Therefore, in such circumstances PCR-based detection is a better choice for quick and precise diagnosis of the disease. PCR-based detection of theileriosis is used worldwide and has high sensitivity and specificity [13,21]. Roy *et al.*, [22] reported that PCR-based assay detects 0.00008% erythrocytic parasitemia equivalent to 16 piroplasm of *T. annulata*. Therefore, it may have special value in diagnosis of diseases from Banaskantha district or elsewhere in the state/country, which have more number of crossbred cattle and high milk production. Using this technique results can be obtained within 3-4 h after receiving the samples and may help to decide the line of treatment, which will results in rapid decision making to control the disease with cost effective treatment in the shortest duration. As described earlier, this is mainly a disease of high yielding exotic crossbred animals, and if not treated rapidly with effective treatment animal becomes anemic, loss weight, decreased milk yield and in extreme cases may lead to death. This will add ups to the economic burdens to the farmers. Moreover, if confirmatory diagnosis is not made than animals will be treated for some other disease conditions, which will ultimately result in unnecessary inoculation of antibiotics and other drugs to the animals. This will also increase the unwanted economic burden on the animal owner as well there will be problem of unwanted antibiotic/drug residue in milk and milk products and development of antibiotic resistant, which will eventually result in rejection of milk and milk product in the international market, pose human health risk. Therefore, farmers may afford this test for routine screening of herd animals.

## Conclusions

It may be concluded from the present study that the molecular technique (PCR) is more sensitive than microscopic examination for diagnosis of theileriosis. Therefore, it may use for screening of samples suspected for theileriosis and has special value in endemic area like Gujarat, where intensive farming has been increased.

## Authors’ Contributions

HCC designed the work. MVP, BKP, SHR, HHP, SIP, MDS and AGB conducted experiments including molecular technique and microscopic examination. ACP had helped in preparation and submission of manuscript. BSC all supervision of the work and examining manuscript. All authors read and approved the final manuscript.
